# A novel prognostic signature of seven genes for the prediction in patients with thymoma

**DOI:** 10.1007/s00432-018-2770-x

**Published:** 2018-10-17

**Authors:** Qiang Li, Yan-Ling Su, Wei-Xi Shen

**Affiliations:** 1 Department of Oncology, Shenzhen Hospital, Southern Medical University, No. 1333, Xinhu Road, Bao’an District, Shenzhen, 518000 Guangdong Province China; 2 Outpatient Department, National Cancer Center, Cancer Hospital and Shenzhen Hospital, Chinese Academy of Medical Sciences and Peking Union Medical College, Shenzhen, Guangdong Province China

**Keywords:** Gene signature, Thymoma, Prognostic signature, Risk score

## Abstract

**Purpose:**

A thymoma is a tumor arising from the epithelium of the thymus, mostly occurring in the anterior mediastinum. The incidence of this disease is low and research progress in this field is slow. Consequently, there is an urgent need to investigate the correlation between molecular regulation and the prediction of survival and prognosis in patients with thymoma.

**Methods:**

We collected thymoma datasets from The Cancer Genome Atlas (TCGA). Differentially expressed genes (DEGs) of TCGA datasets were then determined using R software. A gene signature was obtained by screening prognostic DEGs from the TCGA datasets using univariate and multivariate Cox survival analysis. The summation of the weighted expression levels was calculated as a risk score, which could then be used to predict the survival rate and prognosis of patients with thymoma. The validity of this model was verified by the analysis of receiver operating characteristic (ROC) curves and area under the curve (AUC).

**Results:**

In total, 297 DEGs were identified from the integrated results of TCGA datasets. A seven-gene signature, along with a regression model of prognostic risk, was obtained by Cox survival analysis. The expression levels of the seven genes were then weighted and summed to calculate the risk score for each sample. Patients were effectively divided into high- and low-risk groups using the median risk score (*P* < 0.05). ROC analysis showed that this Cox regression model was effective in predicting the prognosis of patients with thymus tumors (AUC = 0.983, *P* < 0.05).

**Conclusion:**

For the first time, we identified an effective seven-gene signature in patients with thymic tumors. In the future, the risk and prognosis of patients can be preliminarily assessed using this model, although further testing is required to improve rigor.

## Introduction

Thymoma arises from the thymic epithelial cells and accounts for approximately 20% of anterior mediastinal tumors, making this the most common tumor. The annual incidence of thymoma is approximately 0.13/100,000, with the onset age ranging from 30 to 50 years (Strobel et al. 2010; Girard et al. 2009). Research has shown that there is no significant difference in the incidence of thymoma when compared between men and women (Panarese et al. 2014; Shelly et al. 2011). The pathogenesis of thymoma remains unclear, even though a few of the reported cases have suggested that this disease might be related to Epstein–Barr virus or type I T-cell virus infections (Okumura et al. 2002; Lee 2009; Ciardiello and Tortora 2008; Cappuzzo et al. 2005; Erkmen et al. 2011).

The prognosis of patients with thymoma is largely determined by the thymoma histological type, which is complex. This complexity has led to the present lack of uniform measurement standards. The classification of thymoma by the World Health Organization (WHO) is largely based on the proportion of lymphocytes in the thymus tumor. WHO classification type A (medullary type) and type B1 are considered to be less invasive, while type B3 is considered to be more invasive (Cappuzzo et al. 2005; Henley et al. 2004; Gumustas et al. 2013). The criteria for Masaoka staging are based on whether the thymic tumor envelope is infiltrated or whether there is metastasis into other parts of the body. Studies have shown a good correlation between Masaoka staging and the WHO pathological classification, and these classification systems have been widely used in clinical practice as an independent predictor of patient prognosis and survival. Type A (medullary type) and AB (mixed type) thymoma in the WHO classification correspond to Masaoka stages I and II due to less local infiltration. Type B (cortex) thymoma is associated with frequent invasion and metastasis and corresponds to Masaoka stages III and IV (Henley et al. 2002; Luo et al. 2016).

Currently, surgical resection is still the primary treatment for thymoma. Radiation and chemotherapy are effective supplemental treatments (Attaran et al. 2012). However, the treatment of thymus tumors composed of different tissue types still lacks effective guidelines, partly due to histological diversity and partly because of its low incidence.

With recent developments in molecular biology, an increasing number of research projects have begun to investigate methods to accurately predict the prognosis of thymoma, and to develop more effective treatment methods. Studies have shown that the increased expression of tumor-associated genes, such as FPGS (folylpolyglutamate synthase)/GGH (gamma-glutamyl hydrolase) and VEGF (vascular endothelial growth factor), are related to the degree of malignancy in thymic carcinoma and B3 thymoma.^11, 15^ Studies have also shown that c-kit expression is significantly lower in thymic adenoma compared to thymic carcinoma; C-kit expression was detectable in approximately 70–86% of patients with thymic carcinoma, compared to only 0–5% of thymic adenomas. Thus, c-kit could be used as a specific biomarker for thymic carcinoma, as well as a potential target for TKI (tyrosine kinase inhibitor) treatment (Henley et al. 2004; Badve et al. 2012). Several studies have also investigated the epigenetics of thymoma genes, including histone modification, chromatin recombination, and gene methylation (Gumustas et al. 2013; Luo et al. 2016; Badve et al. 2012).

Due to the lack of standardized risk evaluation criteria for patients with thymoma with which to predict prognosis, our study used mRNA-seq datasets from The Cancer Genome Atlas (TCGA). A gene signature for the prognosis of patients with thymoma was then constructed by univariate significance analysis of gene expression and Cox regression survival analysis. The final regression model represents a potentially useful tool but needs to be verified in clinical practice. Nevertheless, our findings will help to elucidate the pathogenesis of thymoma in the future.

## Methods

### TCGA dataset processing and screening for differentially expressed genes (DEGs)

Thymoma genome-wide mRNA-seq expression data, and clinical materials, were obtained from the TCGA website (https://portal.gdc.cancer.gov/). Integration processing of datasets was implemented using Perl (http://www.perl.org/) scripting tools. Gene expression levels in tumor and normal tissues were then compared to identify DEGs (defined as a fold change > 2, *P*_adj_ = 0.01). Perl scripting tools were then used to merge data on clinical survival time and the expression of DEGs. Specific genes associated with survival prognosis were then determined by univariate analysis (*P* < 0.05).

### Kyoko Encyclopedia of Genes and Genomes (KEGG) enrichment analysis and signaling pathway visualization

The KEGG is a database in which the advanced functions and utility of biological systems (cells, organisms, ecosystems) can be investigated using large-scale molecular datasets generated by genome sequencing and other high-throughput experimental techniques (Kanehisa and Goto 2000). In the present study, pathway enrichment analysis was performed on the identified DEGs using the KEGG database (*P* < 0.05, counts = 154). DEGs were converted into an Entrez ID with the help of the RSQLite R package program (https://cran.r-project.org/). Subsequently, the KEGG signaling pathways were coordinated by R package components such as ClusterProfiler, Pathview (http://www.bioconductor.org/) and Stringi (https://cran.r-project.org/). Cytoscape software (http://www.cytoscape.org/) was then used to convert the enriched analysis data into visual images.

### Gene expression signature and risk score evaluation

The composition of the gene expression signature was significantly correlated with the survival time of patients. We were able to calculate the risk score for each patient. Risk score was defined as the summation of weighted mRNA expression levels based on the multivariate Cox regression analysis. The results were as follows: ${\text{risk score}}=\Sigma {\beta _{{\text{genei}}}} \times {\text{Ex}}{{\text{p}}_{{\text{genei}}}}\left( {i={\text{1}} - N} \right)={\beta _{{\text{gene1}}}} \times {\text{Ex}}{{\text{p}}_{{\text{gene1}}}}+{\beta _{{\text{gene2}}}} \times {\text{Ex}}{{\text{p}}_{{\text{gene2}}}}+{\text{ }} \cdots {\text{ }}+{\beta _{{\text{geneN}}}} \times {\text{Ex}}{{\text{p}}_{{\text{geneN}}}}.$ The higher the score, the higher the risk of death. Patients were divided into high- and low-risk groups according to the median risk score and a survival curve was generated (*P* < 0.05). We used the receiver operating characteristic (ROC) curve to assess the classification performance of the gene signature and evaluate its accuracy and specificity. The ROC curve was plotted and the area under the curve (AUC) was estimated using the survival ROC R package components (https://cran.r-project.org/). Next, we used the resultant heat map to show the distribution of the risk score across all patients for each gene.

### Statistical analysis

All data analysis in this study was handled using the R package software (version 3.4.4) and Perl scripting tools. Bioconductor (http://bioconductor.org/) provides data packets for download as a public bioresource site. Continuous variables are described as means and standard deviations, while categorical variables are described as frequencies and percentages. For continuous variables, we used the independent Student’s *t* test and analysis of variance (ANOVA). For categorical variables, we used Pearson’s Chi-squared test and Fisher’s exact tests to detect statistical differences. Next, we used univariate and multivariate Cox regression models, along with survival R packages, to determine predictors of overall survival (OS). The survival ROC R package was used to evaluate the validity of the regression model, and the Pheatmap R package tool (https://cran.r-project.org/) was used to show the distribution of patient risk degree. For OS analysis, any cause of death was defined as an event, and survivors were defined as censored events. All *P* values were bilateral and *P* < 0.05 was considered significant.

## Results

### Workflow presentation

**Fig. 1:**
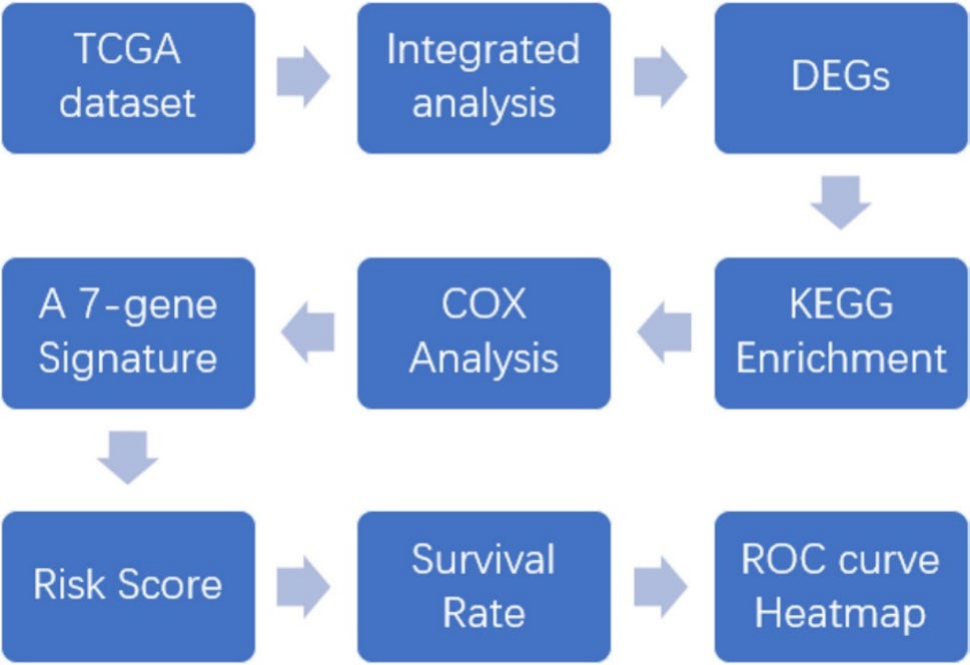
Development and validation of the seven-gene signature. Genomic mRNA-seq expression data and clinical thymoma materials were downloaded from the TCGA database website. KEGG pathway enrichment was performed on 297 DEGs screened for differential expression analysis. The seven-gene signature was developed by univariate and multivariate Cox regression analysis (*P* < 0.05). This regression model was validated using the TCGA datasets

Our seven-gene signature was developed and validated using a series of combination processing (Fig. 1). Genomic mRNA-seq expression data and clinical thymoma materials were downloaded from the TCGA database website. Standardized datasets were obtained through annotation and integration. We used the R package software (version 3.4.4) to compare gene expression levels in tumor and normal tissues, and identified DEGs in all samples. DEG enrichment analysis was performed in the KEGG database, and the signaling pathway characteristics of DEGs were demonstrated by visualization graphics. A Perl script tool was used to merge clinical survival time and DEG expression, and univariate analysis was used to determine specific genes associated with survival prognosis (*P* < 0.05). Based on this, we developed a seven-gene signature model via multivariate Cox analysis. The sum of the weighted expressions of the seven-gene signature was then calculated as a risk score. The median risk score was then used to divide patients into high- and low-risk groups (*P* < 0.05). The survival curve was clearly able to demonstrate the OS rate. The ROC curve and AUC verified that the Cox regression model had good validity.

### Characteristics of the TCGA datasets

The mRNA-seq expression datasets collected from the TCGA database contained 121 samples from 119 patients diagnosed with thymus tumors, and consisted of 62 men and 57 women. Of these, 99 were Caucasians, 12 were Asians, and 8 were from other backgrounds. The age at thymoma diagnosis ranged from 17 to 84 years. Nine of the 119 patients had died, ranging from a total of 124–3,488 days since death. Of the 121 samples, 2 were normal and 119 contained tumors. TCGA datasets were classified using transcriptome profiling data categories, gene expression quantification of data types, and high-throughput sequencing (HTseq) counts of workflow type.

### KEGG enrichment analysis and signaling pathway visualization

**Fig. 2:**
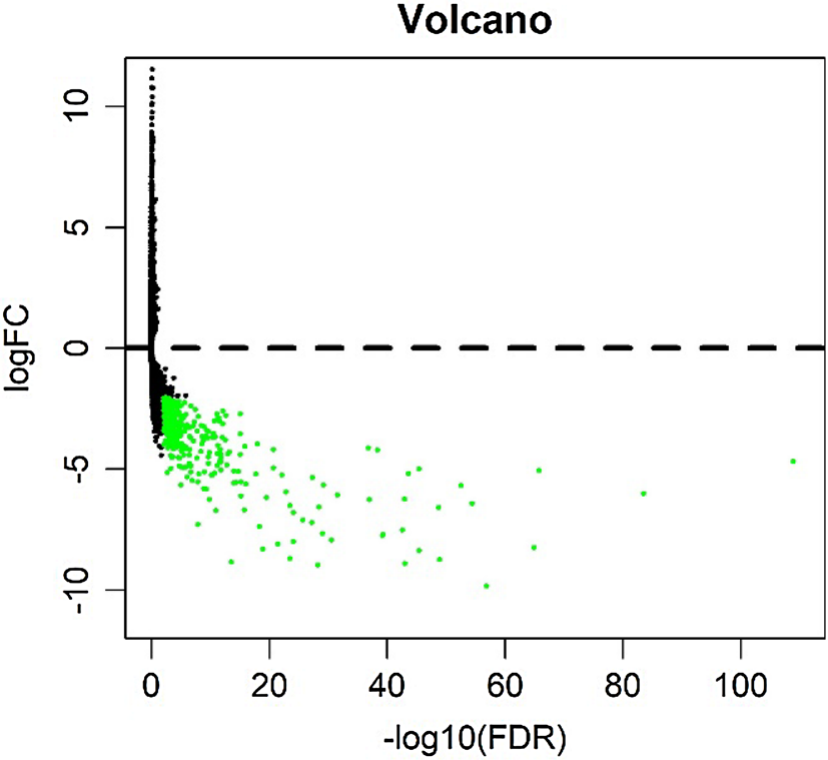
Volcano plot of differentially expressed genes (DEGs). We detected 297 DEGs in thymic tissues from normal samples; each green dot shows a downregulated gene (fold change > 2, *P*_adj_ = 0.01)

**Fig. 3:**
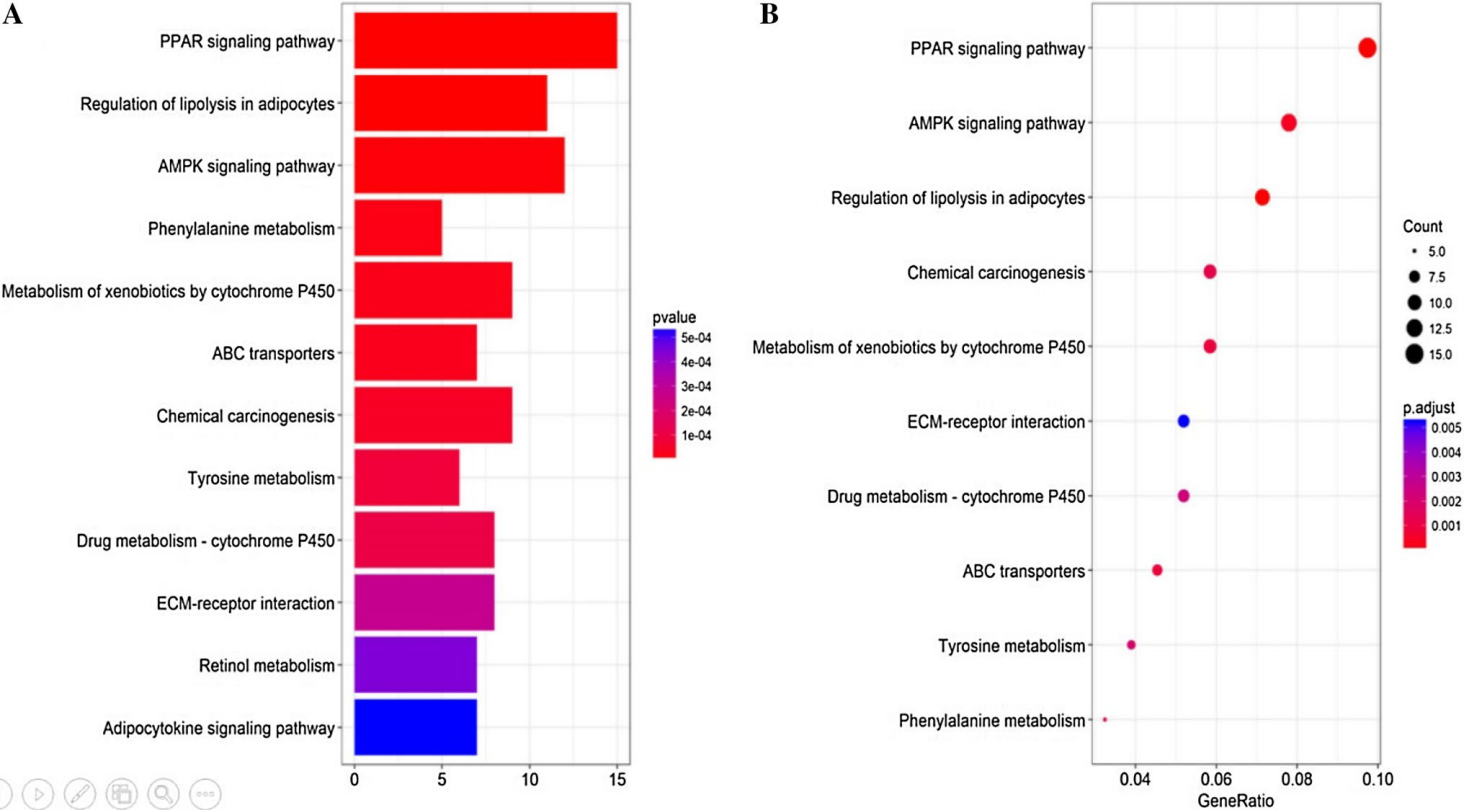
Enrichment analysis performed on DEGs using the KEGG database. **a** Bar plot showing the top 12 KEGG pathways arranged in order of *p* value. The correlation is more significant as the red/blue ratio increases (*P* < 0.05, counts = 154). **b** Dot plot showing the top 10 KEGG pathway permutations according to the number of DEGs. The correlation is more significant as the red/blue ratio increases (*P* < 0.05, counts = 154)

**Fig. 4:**
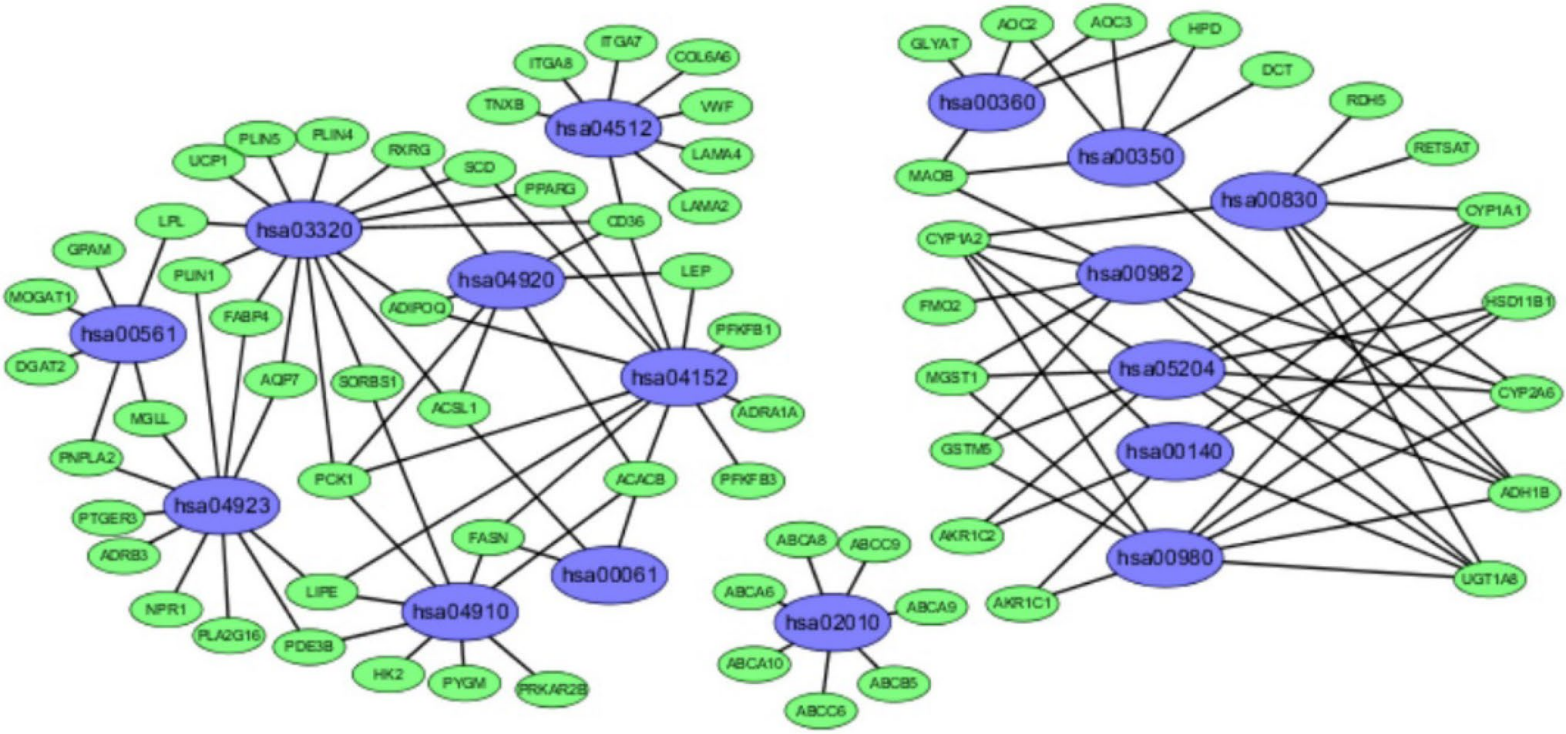
Enrichment analysis data (as visualized by Cytoscape) showing 16 KEGG functional pathways. All of the enriched genes were downregulated rather than upregulated

We analyzed the differential expression of 121 samples in the TCGA datasets and identified 297 DEGs, all of which were downregulated genes (Fig. 2). Signaling pathway enrichment analysis of the DEGs was then performed using the KEGG database. In total, 154 DEGs were significantly associated with 16 signaling pathways (*P* < 0.05, counts = 154) (Table 1). The top 5 listed KEGG signaling pathways were as follows: hsa03320 (PPAR signaling pathway); Hsa04923 (regulation of lipolysis in adipocytes); Hsa04152 (AMPK signaling pathway); Hsa00360 (phenylalanine metabolism) and Hsa00980 (metabolism of xenobiotics by cytochrome P450). Subsequently, KEGG signaling pathway diagrams were generated using R package components such as ClusterProfiler, Stringi and Pathview (Fig. 3a, b). Enrichment analysis data were visualized using Cytoscape software (Fig. 4).

**Table 1:** Enrichment analysis of differentially expressed genes (DEGs) in the KEGG pathways

ID	Description	Gene ratio	*P* value	Adjusted *P* value	Count
hsa03320	PPAR signaling pathway	15/154	1.79E-11	3.39E-09	15
hsa04923	Regulation of lipolysis in adipocytes	11/154	9.42E-09	8.90E-07	11
hsa04152	AMPK signaling pathway	12/154	6.27E-06	0.000395	12
hsa00360	Phenylalanine metabolism	5/154	1.81E-05	0.000857	5
hsa00980	Metabolism of xenobiotics by cytochrome P450	9/154	2.43E-05	0.000908	9
hsa02010	ABC transporters	7/154	2.88E-05	0.000908	7
hsa05204	Chemical carcinogenesis	9/154	4.50E-05	0.001214	9
hsa00350	Tyrosine metabolism	6/154	8.37E-05	0.001978	6
hsa00982	Drug metabolism—cytochrome P450	8/154	0.00011	0.002318	8
hsa04512	ECM-receptor interaction	8/154	0.000276	0.005215	8
hsa00830	Retinol metabolism	7/154	0.000439	0.007549	7
hsa04920	Adipocytokine signaling pathway	7/154	0.000527	0.008294	7
hsa00140	Steroid hormone biosynthesis	6/154	0.001309	0.019025	6
hsa00561	Glycerolipid metabolism	6/154	0.001559	0.021044	6
hsa04910	Insulin signaling pathway	9/154	0.002091	0.025314	9
hsa00061	Fatty acid biosynthesis	3/154	0.002143	0.025314	3

### Seven-gene signature and risk score development

Next, we screened the 297 DEGs by univariate survival analysis and Cox regression analysis. This revealed a significant correlation between seven genes as independent predictors of OS (*P* < 0.05). This seven-gene signature involved LIPE, FBLN2, ABCA10, DGAT2, SLC16A7, SELENBP1, and KLF4. Of these, LIPE, FBLN2, and KLF4, were protective factors while the rest were risk factors. This seven-gene signature is of great importance for the prognostic evaluation of thymus tumors. Each patient’s risk score was calculated based on summation of the weighted expression of these 7 genes (Table 2) (likelihood ratio test = 33.34, *P* = 2e−05, *n* = 118), as follows: risk score = *Σβ*_genei_ × Exp_genei_ (*i* = 1–7) = −0.929 × Exp_LIPE_ + − 1.611 × Exp_FBLN2_ + 1.513 × Exp_ABCA10_ + 1.908 × Exp_DGAT2_ + 1.417 × Exp_SLC16A7_ + 1.045 × Exp_SELENBP1_ + − 0.781 × Exp_KLF4_. The higher the risk score, the worse the clinical prognosis. Next, patients were divided into high- and low-risk groups using their median risk score. Using this Cox regression model, it was possible to accurately predict survival.

**Table 2:** A seven-gene signature identified by multivariate Cox regression analysis

	Coef	Exp (Coef)	SE (Coef)	*z*	*p*
LIPE	− 0.929	0.395	0.403	− 2.3	0.0213
FBLN2	− 1.611	0.2	0.541	− 2.98	0.0029
ABCA10	1.513	4.538	0.692	2.19	0.0289
DGAT2	1.908	6.742	0.651	2.93	0.0034
SLC16A7	1.417	4.123	0.633	2.24	0.0251
SELENBP1	1.045	2.842	0.366	2.86	0.0043
KLF4	− 0.781	0.458	0.551	− 1.42	0.1565

Likelihood ratio test = 33.34, *p* = 2e-05, *n* = 118, number of events = 9

### Validation of TCGA datasets

**Fig. 5:**
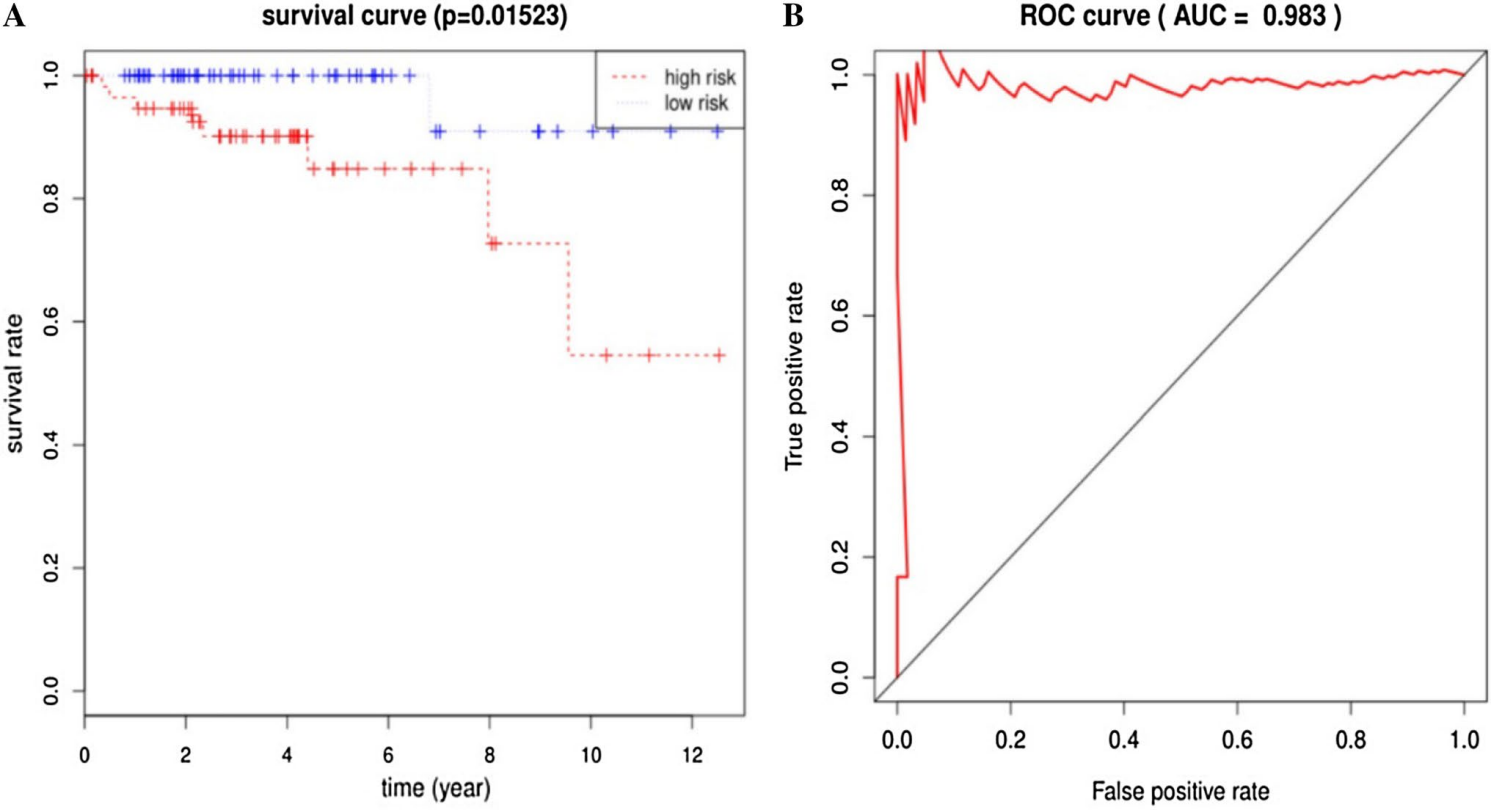
Validation of the seven-gene signature. **a** Risk score was significantly correlated with overall survival (OS). Risk score was an independent prognostic factor for OS in multivariate Cox regression analysis (*P* = 0.01523). The median score divided patients into high- and low-risk groups. The 5-year survival rate of patients in the high-risk group was 0.848% (standard error: 0.0651; 95% confidence interval: 0.730, 0.986). **b** ROC validation of the seven-gene signature. The area under the curve (AUC) was 0.983 (*P* < 0.05), thus demonstrating that the seven-gene signature had high sensitivity and specificity for the classification of thymoma from normal controls

**Fig. 6:**
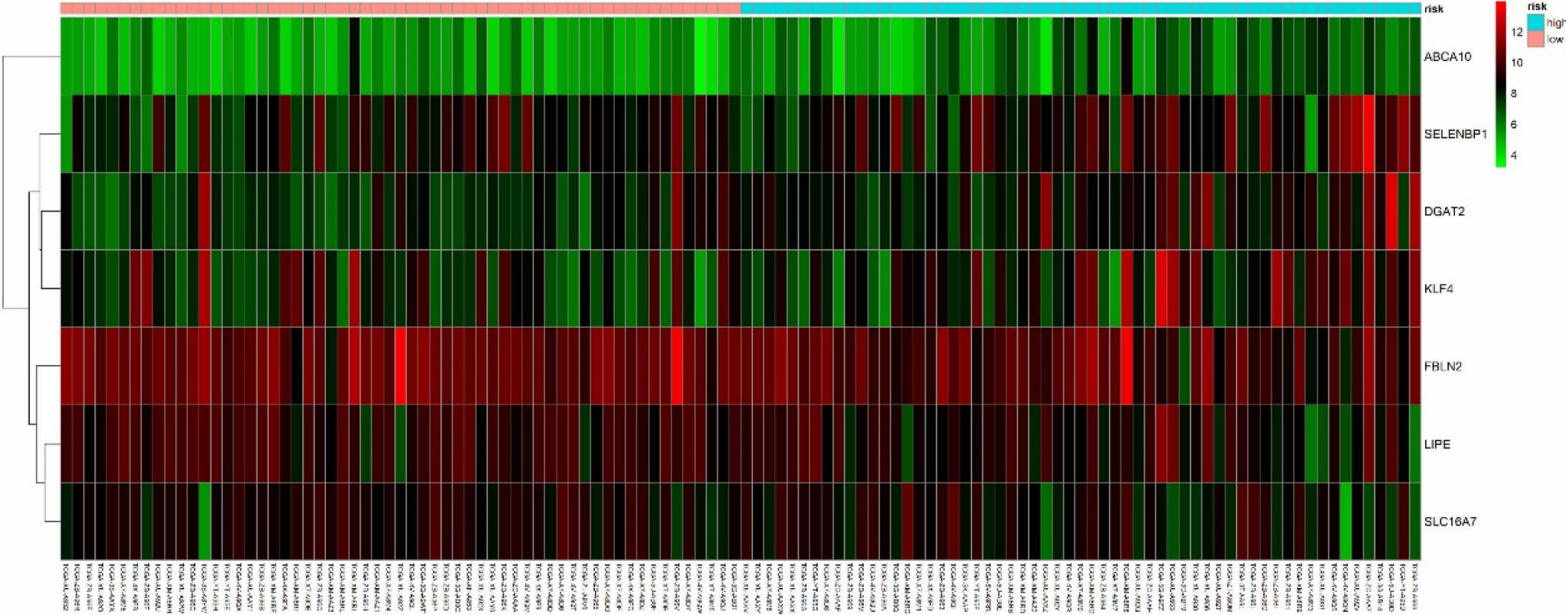
Heatmap of the seven-gene signature in TCGA datasets. Each column represents a sample and each row represents one of the seven genes. The expression levels of the seven genes are shown in different colors, from orange to blue with increasing risk

Next, the risk score for the seven-gene signature was validated using the TCGA datasets. Each patient was assigned to a high- or low-risk group based on their risk score. We found that risk score was significantly associated with OS as a prognostic factor in univariate analysis (*P* < 0.05). Analysis also revealed that risk score was an independent prognostic factor of OS in multivariate Cox regression analysis, which included clinical pathological features (*P* < 0.05) (Fig. 5a). ROC curve and AUC (= 0.983) analysis showed good sensitivity and specificity (*P* < 0.05) (Fig. 5b). A cluster for the seven-gene signature was then determined using heat mapping of the risk scores for all patients (Fig. 6). These validation results showed that the seven-gene signature showed good performance for the normal control thymoma samples in terms of classification.

## Discussion

Thymoma is a tumor formed by the abnormal differentiation of thymic epithelial cells; the highest incidence of this abnormal differentiation occurs in the anterior mediastinum. The incidence of thymoma is approximately 0.13/100,000 and mostly affects patients aged 30–50 years old, without gender difference (Strobel et al. 2010; Girard et al. 2009; Panarese et al. 2014). The pathogenesis of thymoma remains unclear, although some studies have suggested that it might be associated with certain viral infections. However, the low incidence of thymoma has limited the development of large-scale clinical trials and in-depth basic research (Okumura et al. 2002; Lee 2009; Kelly 2013; Riely and Huang 2010).

The histological features of thymus are variable. In the WHO classification, the proportion of lymphocytes in thymic tissues generally increases gradually from type A to type B3. The stepwise change in lymphocyte composition is known to be associated with an increase in the degree of tumor malignancy (Henley et al. 2002; Marx et al. 2014). Studies have also shown a good correlation between Masaoka staging and WHO classification; types A and AB in the WHO classification correspond to stages I and II of the Masaoka classification while type B in the WHO classification corresponds to Masaoka stages III and IV. The 10-year survival rate of patients with type A thymoma can reach 100%. Type C, also known as thymic carcinoma, has a median survival duration of 24–49 months and a 5-year survival rate of 30–50% (Henley et al. 2002; Sasaki et al. 2002). However, at present, there are still no unified evaluation standards for thymus tumors with different histological features.

With recent developments in molecular biology, an increasing number of techniques are been applied to investigate tumor genome, such as transcriptome analysis, gene mutation detection and epigenetic analysis (Yao et al. 2017; Mao et al. 1998). Based on TCGA database mining, our present study integrated and analyzed thymoma datasets to identify DEGs in tumor tissues. We then identified a seven-gene signature and risk score by univariate and multivariate Cox regression analysis; risk score was significantly correlated with OS. Consequently, this system could predict the OS rate of patients with thymoma in an effective manner. Furthermore, our seven-gene signature may be useful in elucidating the pathogenesis of thymus tumors. Our results represent the first description of the genetic signature of thymus tumors.

Within the identified gene signature, all seven genes were significantly associated with the prognosis of thymoma. Of these, LIPE, FBLN2, and KLF4 were found to be protective factors. LIPE hydrolyzes stored triglycerides to produce free fatty acids and can also convert cholesterol esters into free cholesterol to produce steroid hormones. The mRNA levels of LIPE have been shown to be significantly increased in the adipose tissue of cancer patients (Gumustas et al. 2013; Thompson et al. 1993). FBLN2 is located on chromosome 3p25.1; the binding of FBLN2 to fibronectin and other ligands depends on calcium. This protein can also act as an adapter to coordinate interactions between FBN1 and ELN. FBLN2 is associated with the occurrence and development of tumors by interaction with extracellular matrix (ECM) proteins (Law et al. 2012; Falkson et al. 2009). KLF4 is an activated or repressible transcription factor that regulates the expression of key transcription factors during embryonic development and plays an important role in maintaining embryonic stem cells and preventing their differentiation (Kelly 2013; Zhang et al. 2010).

We also investigated the molecular function of the remaining risk factors. ABCA10 is made up of 40 exons that are commonly expressed in the heart, brain, and gastrointestinal tract. The expression of ABCA10 is inhibited by the introduction of cholesterol into macrophages, indicating that it is a cholesterol-response gene and involved in the maintenance of macrophage lipids in a steady state (Wenzel et al. 2003). The DGAT2 gene is located on chromosome 11q13 and, as a key enzyme in fat metabolism, is a candidate obesity gene. The DGAT2 protein is a glycolipid metabolism component involved in the triglyceride biosynthesis pathway (Friedel et al. 2007). SLC16A7 (also known as monocarboxylate transporter protein 2, MCT2) is mainly located in the peroxisomes of prostate cancer cells and interacts with Pex19 via the peroxidase transport mechanism. The overexpression of SLC16A7 in malignant cells, compared to non-malignant cells, is directly related to peroxidase localization. SLC16A7 is significantly overexpressed in malignant prostrate tumors, suggesting that it may represent a biomarker of prostate cancer (Valenca et al. 2015). SELENBP1 was previously identified as the most significantly downregulated protein in ovarian cancer cells by membrane proteomics analysis. Selenium can interfere with the androgen pathway, which is regulated by the expression of SELENBP1. In malignant ovarian cancer, changes in SELENBP1 expression are useful indicators of abnormal selenium/androgen pathways, and such changes may reveal prognostic information relating to ovarian cancer (Huang et al. 2006).

Our present study has several limitations which need to be taken into consideration. Firstly, we identified our seven-gene signature based solely on bioinformatic analysis. Further experiments are now needed to verify these results. Secondly, in addition to the TCGA datasets, it is necessary to mine other databases to provide further validation. Thirdly, the seven-gene signature model identified in this study needs to be closely integrated with clinical practice. To improve these deficiencies, we plan to collect thymic tumor samples, and clinical prognosis information, and then use specific experiments to verify our results. It is now necessary to conduct more studies on the functional effects of these seven genes and their relevance to patient survival; this is important because we have only limited functional knowledge of the seven genes in the signature identified.

## Conclusion

In summary, our study suggested that our seven-gene signature could be used as a prognostic indicator for thymoma with which to predict patient risk. This signature could be applied to clinical practice in an effective manner. Further research is now needed to confirm these results.
